# Case of Reduction of Dislocation of Femur after the Plan Discovered by Dr. Reid

**Published:** 1851-10

**Authors:** A. Barnard

**Affiliations:** Adrian, Mich.


ART. IV.—*Case of Reduction of Dislocation of Femur after the plan discovered by Dr. Reid.* By A. BARNARD, M. D., of Adrian, Mich.

In noticing an essay on Dislocation of the Femur on the Dorsum Ilii, delivered before the Monroe County Medical Society, in the August number of the Journal, by W. W. Reid, I was forcibly impressed with the plan as a good one. Having never met with a case of the kind, and withal dreading the idea of being called to one, I gave the article a close examination. But a few days since I was summoned to see Stephen Fowler, about three miles from our village, who was shoveling sand near the railroad. It caved from over head, and buried him up. He was soon extricated and brought to the nearest house. By some means, probably by one corner of the shovel, the scrotum was lacerated, and let out one of the testes — a small affair compared to a luxation of the Femur on the Dorsum Ilii, which I discovered on examination. I cannot say that I was in ecstacies exactly, neither obliged to him for giving me an opportunity to reduce it, because I was not quite so certain of succeeding as Dr. Reid. I called to my assistance Dr. Kimball, and on getting him under the influence of chloroform and ether (about a quarter part of the former to three-quarter parts of the latter) made use of the manipulations suggested by Dr. Reid, and in thirty seconds succeeded in reducing it — then, indeed, we were in ecstacies. A more splendid improvement, in the reduction of dislocated femur, probably can never be.

If the above will be of any benefit to the readers of the Journal, or to suffering humanity, and I believe it most assured will, you are at liberty to give it a place in the Journal.

